# Neurodevelopmental profile in Angelman syndrome: more than low intelligence quotient

**DOI:** 10.1186/s13052-016-0301-4

**Published:** 2016-10-21

**Authors:** S. Micheletti, F. Palestra, P. Martelli, P. Accorsi, J. Galli, L. Giordano, V. Trebeschi, E. Fazzi

**Affiliations:** 1 Unit of Child Neuropsychiatry and Early Neurorehabilitation, ASST Spedali Civili, Piazzale Spedali, Civili 1, 25123 Brescia, Italy; 2 Cognition Psychology Neuroscience Lab, University of Pavia, Pavia, Italy; 3 Department of Clinical and Experimental Sciences, University of Brescia, Brescia, Italy; 4 Unit of Child Neurology and Psychiatry, ASST Spedali Civili, Piazzale Spedali Civili 1, 25123 Brescia, Italy

**Keywords:** Angelman syndrome, Motor impairment, Neurovisual disorder, Intellectual disability, Communicative disorders

## Abstract

**Background:**

Angelman Syndrome (AS) is a rare neurodevelopment disorder resulting from deficient expression or function of the maternally inherited allele of UBE3A gene. The aim of the study is to attempt at providing a detailed definition of neurodevelopmental profile in AS, with particular regard to motor, cognitive, communicative, behavioural and neurovisual, features by using standardized instruments.

**Method:**

A total of ten subjects aged from 5 to 11 years (4 males and 6 females) with molecular confirmed diagnosis of AS (7 15q11.2-q13 deletion and 3 UBE3A mutation) were enrolled in our study. All of them underwent an assessment protocol including neurological and neurovisual examination and the evaluation of motor (Gross Motor Function Measure Scale), cognitive (Griffiths Mental Development Scale and Uzgiris-Hunt Scale); adaptive (Vineland Adaptive Behavioural Scale); communication (MacArthur-Bates Communicative Development Inventory and video-recordings children’s verbal expression), behavioural aspects (IPDDAG Scale) and neurovisual aspects.

**Results:**

All children presented motor function involvement. A severe cognitive impairment was detected with different profiles according to the test applied. In all cases, communicative disability (phonemic inventory, word/gesture comprehension and production) and symptoms of inattention disorder were revealed. Neurovisual impairment was characterized by refractive errors, fundus oculi anomalies, strabismus and/or oculomotor dysfunction.

**Conclusion:**

AS presents a complex neurodevelopmental profile in which several aspects play a negative role in global development leading to a severe functional impairment. Intellectual disability is not the only component because neurovisual functions and behavioural disorders may worsen the global function and are needed of specific rehabilitation programs.

## Background

Angelman syndrome (AS) is a rare neurodevelopment disorder with resulting from deficient expression or function of the maternally inherited allele of UBE3A gene on chromosome 15, which plays an important role in the cellular ubiquitin-proteasome pathway and synaptic development [1].

Four genotypic mechanisms that confer the AS phenotype are currently known, i.e., deletion or mutation of the maternal UBE3A gene, paternal uniparental disomy, or an imprinting defect [2].

Even if research into genotype-phenotype correlations reveals a more severe impairment among children with deletion forms rather than those with other genetic mechanisms [3, 4], all genetic expressions lead to a similar clinical phenotype [5] characterized by developmental delay, movement or balance disorder, specific behavioural characteristics and speech impairment [6].

The behavioural characteristics of AS are present in all patients irrespective of the type of genetic abnormality and often prompt clinicians to consider the diagnosis [7]. Apparent happiness is the hallmark of the syndrome, associated with profuse smiling, poorly specific laughing and general exuberance, with hyperactivity, stereotypes, and proactive social contact [8].

Cognitive delay is usually evident within the first year of life [9] and in the few studies that applied psychometric assessments most patients reach plateau at a developmental level of 24-30 months [10–12]. 80–90 % of AS patients are reported to have epilepsy [13]. In particular atypical absence, myoclonic, generalized tonic–clonic, and atonic seizures [14].

Truncal hypotonia and distal extremity hypertonia/hyperreflexia characterize the neurological examination of children with AS [15]. Movement disturbances such as jerkiness, ataxic gait and tremors are specific; abnormalities of tone and impaired balance contribute to the delayed acquisition of motor skills (sitting after 12 months, walking between 2 and 6 years) [9].

Communication difficulties are prominent features in AS. Most patients do not acquire expressive speech or have a vocabulary of few words [12]; some can communicate using sign language and others can use gesture or augmentative communication devices [7, 9]. Common language assessment tools are difficult to apply and receptive-expressive skills have been mostly analysed by parents’ reports [12, 16].

As regards daily living skills only few studies have applied standardized instruments to explore adaptive behaviour profiles in AS [4, 11, 17]. Peters and colleagues [11] proposed to parents the Vineland Adaptive Behaviour Scales (VABS) and found a relative strength in socialisation and a weakness in motor skills in a group of 20 patients aged 5 months–10 years. Brun Gasca and colleagues [18] proposed the Inventory for Client Agency in a group of 25 children with AS aged 1–17 years and found highest scores in personal living skills and lowest for social and communication skills.

Poor data are also reported on visual problems in AS population. Ocular problems in AS include refractive errors (usually hypermetropia and astigmatism), iris and choroidal hypopigmentation, and esotropia or exotropia [19, 20]. They are more common in those with deletion. Nystagmus is reported but is not common.

Despite many descriptive data documenting the cognitive, linguistic and behavioural profiles of AS, no studies, to our knowledge, provide a comprehensive description of the clinical profile of AS collecting and relating different developmental areas such as motor, neurovisual, linguistic, cognitive, adaptive and behavioural features.

The aim of the study was to analyse these neurodevelopmental areas that concur in the development of AS patients, and to describe a specific neurodevelopmental clinical profile which can be the basis to promote tailored early intervention programs.

## Methods

### Participants

All the children at scholar age (5 to 11 years) with a molecular confirmed diagnosis of AS, referred to our Department between 2013 and 2015, have been enrolled. Exclusion criteria were the presence of epilepsy not controlled by antiepileptic drugs and the presence of intercurrent diseases. Ten subjects were recruited: 4 males and 6 females, age ranging from 5 years and 2 months to 11 years (mean age 7 years and 1 month; SD 1 year and 7 months).

Demographic, genetic and clinical characteristics are reported in Table 1.

**Table 1:** Clinical characteristics of the sample

Genetic anomaly	Patient	Age	Sex	Age at diagnosis	Pregnancy	GA	Perinatality
UBE3A mut	Pat.1	8 yr 11 mo	M	34 mo	Normal	Term	Normal
	Pat. 2	5 yr 2 mo	F	35 mo	Normal	Term	Normal
	Pat. 3	8 yr 1 mo	F	23 mo	Normal	Term	Normal
Deletion	Pat. 4	5 yr 3 mo	M	11 mo	Normal	Term	Normal
	Pat. 5	6 yr 5 mo	M	9 mo	Gestosis	Preterm	C section
	Pat. 6	10 yr	M	13 mo	Normal	Term	Normal
	Pat. 7	6 yr 7 mo	F	10 mo	Normal	Term	Normal
	Pat. 8	8 yr 2 mo	F	22 mo	Normal	Term	Normal
	Pat.9	6 yr 4 mo	F	36 mo	Normal	Term	Normal
	Pat. 10	3 yr 11 mo	F	21 mo	IUGR	Term	Normal

*yr* years, *mo* months, *M* male, *F* female, *IUGR* intrauterine growth retardation

In 7 children UBE3A deletion (DEL) was detected and in 3 patients a UBE3A mutation (MUT) was present. The age at the diagnosis varied according to the type of genotypic mechanism: patients with DEL were diagnosed between 9 and 36 months (mean 17.4 months SD 9.7), those with MUT were diagnosed later (mean 30.6, SD 6.7). Birth weight (mean 2931 gr, SD 428 gr) and head circumference (mean 33.5 cm; SD 1.15 cm) at birth were within normal range in 9 patients. On the contrary, 5 patients presented a head circumference below third centiles and 4 subjects had weight and height below normal range at the moment of protocol submission.

All children except two had epilepsy and needed antiepileptic drugs (valproic acid in 2 patients, associated with etosuccimide, levetiracetam or clobazam in 5 patients, clonazepam in 1 patient). 7 patients have started antiepileptic therapy since they were younger than 2 years old. One patient started therapy when she was 3 years old. All patients were seizure-free.

Neuro-imaging was normal in 3 subjects, in 5 subjects mild periventricular anomalies were detected. Two children did not undergo neuroimaging exams because of parents’ refusal.

### Procedure

An evaluation protocol including motor abilities, cognitive and adaptive functions, language and communication skills, behavioural aspects and neurovisual functions was proposed to all families. Evaluation was composed both by clinical assessments and by parents’ questionnaires. Ethical Committee of ASST Spedali Civili in Brescia, Italy, approved the study and informed consent was obtained from parents.

Motor function was evaluated by the collection of anamnestic data especially about motor milestones, Gross Motor Function Measure Scale (GMFMS) [21] and neurological examination.

Because of the difficulties to propose intelligence scales according to chronological ages (massive presence of floor effects on intelligence tests such as Leiter-r scale) [22], all the children were assessed by using Griffiths Mental Developmental Scales (GMDS) [23] and Uzgiris Hunt Scale (UHS) [24], based on Piaget’s theory of intelligence development [25]. VABS [26] were proposed to the parents to evaluate adaptive functions.

Because of the subjects’ reduced compliance on standardized language tests, communication and receptive/expressive language abilities were analysed by the Italian version of the MacArthur-Bates Communicative Development Inventory (PVB) [27], and the video-recordings children’s verbal expression during a structured child playing observation. PVB was proposed to compare action/gesture production, word comprehension/production and the relationship between these three domains. An investigation into phonemic inventory was obtained by recording and analysing child speech production during semi-structured playing observation.

The Italian questionnaire IPDDAG [28] was proposed, according to patient’s mental age (MA) to investigate behavioural aspects, particularly those referred to Attention Deficit and Hyperactive Disorder (ADHD) symptoms.

All participants underwent a clinical and neurovisual assessment [29] that includes the evaluation of ophthalmological aspects (refraction under cycloplegia, anterior segment and fundus oculi), oculomotor components (visual axis alignment to detect strabismus, extrinsic ocular motility, fixation, smooth pursuit, saccades, abnormal ocular movements), perceptual visual elements (visual acuity, contrast sensibility, optokinetic nystagmus and visual field) and visual attention.

## Results

### Motor and physical evaluation

Table 2 details motor abilities in the sample.

**Table 2:** Motor development and GMFMS evaluation results

		Head control	Sitting	Walking	GMFMS					
					LR	Si	CK	St	WRJ	TS
UBE3A mut	Pt 1	7 mo	9 mo	30 mo	100	100	88	82	65	87
	Pt 2	5 mo	10 mo	24 mo	100	98	88	77	36	80
	Pt 3	4 mo	16 mo	23 mo	100	100	95	82	49	85
	Mean	5 mo	12 mo	26 mo	100	99	91	81	50	84
Deletion	Pt 4	4 mo	15 mo	36 mo	100	100	85	82	43	82
	Pt 5	4 mo	10 mo	48 mo	100	100	83	80	40	80
	Pt 6	5 mo	13 mo	24 mo	100	100	100	77	43	84
	Pt 7	9 mo	24 mo	48 mo	100	100	88	33	17	68
	Pt 8	8 mo	19 mo	72 mo	100	97	31	46	25	60
	Pt 9	10 mo	24 mo	48 mo	100	100	90	46	28	73
	Pt 10	5 mo	8 mo	30 mo	100	100	87	40	85	19
	Mean	6 mo	16 mo	43 mo	100	99	80	57	34	66
Total	Mean	6 mo	14 mo	38 mo	100	99	83,5	64,5	43	72

*mo* months, *LR* Lying and Rolling, *Si* sitting, *CK* crawling and kneeling, *St* standing, *WRJ* walking, running and jumping, *TS* total score

Anamnestic data revealed that all the subjects presented delay in motor milestones (mean age head control 6.9 months, SD 2.8; sitting 16.2 months, SD 6.3; walking 38.3 months, SD 15.6), also confirmed by GMFMS, which showed a homogeneous profile among the patients with a mild motor impairment in standing ability and a more severe impairment in walking tasks. Children with DEL reached motor milestones later (mean age head control 7.4 months, SD 2.9; sitting 18.1 months, SD 6.4; walking 43.4 months, SD 15, 8) than those with MUT (mean age head control months, sitting 11, 7 months, walking 25, 7 months).

In all the subjects, neurological examination detected ataxia and motor stereotypes, which were associated with tremor/jerky movements in 2 patients, distal hypertonia at lower limbs in 5 patients, hypotonia in 2, scoliosis in 1.

### Cognitive evaluation

Cognitive evaluation with GMDS showed the presence of severe intellectual disability in the whole sample. The definition of mental age (MA) was preferred instead of the measurement of Developmental Quotient. Most of the patients presented in fact severe difficulties which are not effectively described by centiles (all the patients were below the first centile in all the subscales). The mean MA of the total score was 14.1 months (SD 4.7; range 9.3–23). None of the patients reached a MA higher than 24 months. The profile in each subscale was substantially homogeneous. Higher scores were obtained in Locomotor (mean MA 17.3 months; SD 5.6; range 11–27) and Personal and Social subscales (mean MA 15.8 months; SD 6.5; range 8–24.6). Lowest scores characterized Language (mean MA 11.4 months; SD 3.2; range 7.5–15.6) and Performance Scales (mean MA 12.5 months; SD 3.2; range 6.5–21).

Cognitive impairment was more severe in DEL group (mean MA 11.7 months; SD 1.7; range 9.3–17) than in MUT group (mean MA 19.7 months; SD 3; range 17–23).

In order to obtain a qualitative perspective, the sample underwent an evaluation with UHS, which confirmed the presence of a severe intellectual disability. Because of the ceiling effect two patients reached the highest values in the majority of the subscales (see Table 3), showing thus a MA greater than 24 months. Four children mastered cognitive precursors of language, since they reached at least the fifth stadium at the Object Permanence, Means ends and Operational Causality subscales. These were the only children who performed deictic gesture and showed the highest level of word expression.

**Table 3:** GMDS evaluation and communicative and linguistic findings at PVB

		GMDS (MA in months)						PVB (MA in months)				
		M	PS	L	EHC	P	T	WC	WP	AGP	PP	DG
UBE3A mut	Pt 1	20	22	14	22	21	19	>17	<8	17	No	Yes
	Pt 2	27	25	16	24	20	23	>17	14	>17	No	Yes
	Pt 3	25	23	13	17	14	17	17	<8	14	No	Yes
Deletion	Pt 4	19	15	13	13	10	14	11	<8	10	No	No
	Pt 5	8	7	9	8	9	13	12	<8	12	No	No
	P 6	19	11	8	10	6	11	13	<8	10	No	No
	Pt 7	9	8	9	9	9	13	8	<8	9	No	No
	Pt 8	12	10	10	8	9	10	11	<8	11	No	No
	Pt 9	13	12	9	11	9	12	15	<8	21	No	Yes
	Pt 10	16	22	15	19	17	17	13	<8	15	No	Yes

GMDS-R Scales: *M* Motor, *PS* personal social, *L* language, *EHC* eye hand coordination, *P* performance, *T* total

PVB: *WC* word comprehension, *WP* word production *AGP* action/gesture production, *PP* pretend play, *DG* deictic gestures

### Adaptive functions

VABS showed a severe impairment in all the investigated domains. In some scales the majority of the patients showed a floor effect on those scales which imply a level of competences usually reached over 24 months (written, domestic, coping skills, interpersonal relationships subdomains). Thus these scales did not provide reliable information about the real skills of the subjects. The area of Communication skills (receptive, expressive, and written communication skills) was the most affected: 8 patients showed a floor effect (MA <18 months). Daily living domain was also severely affected: 5 patients showed a MA lower than 18 months. A MA of 37 months was reached by a MUT patient who also showed the best scores at cognitive evaluation. In Socialization domain (Interpersonal Relationships, Play and Leisure Time, Coping skills) 3 patients showed the lowest scores (floor effect), the other patients showed competences between 18 and 26 months (mean 21 months; SD 2, 8).

Scores at Motor skills domain were heterogeneous: 4 patients scored lower than 18 months, two children reached a score higher than 24 months. Children with MUT showed better competences in all the adaptive domains. In particular, they presented better performance in personal and gross motor skills.

### Language and communication

The evaluation of word comprehension/production and action/gesture production (routines gestures, actions with objects, pretending play and imitation gestures) revealed a severe impairment of early communication skills. The mean word comprehension MA was 14.1 months (DS 3.7; range 8–18). Word production MA was not evaluated because of the presence of floor effect in the entire sample. Three children could speak up to 5 words.

In most of the cases the difference between the understood and spoken words was extremely high.

The mean action/gesture production MA was 12.5 months (DS 12.5; range 10–17) and the best represented actions/gestures concerned playing/routines activities (mean percentage gesture comprehension: 48 %). None of the patients were able to access to pretending play skills. Four children had deictic gestures and 6 had other referential gestures. As regards phoneme inventory, all patients were able to pronounce the "a" vowel. Consonants were present only in four patients, who could pronounce nasal and stop consonants (see Table 4).

**Table 4:** Phoneme inventory (number of patients who can pronounce consonants/vowels)

Phoneme inventory								
Consonantic inventory								
			m	n	η	p	t	k
Position	Initial	Deletion	1	/	/	1	0	1
		UBE3A mut	1	1	/	1	1	1
	Median	Deletion	1		/	1	1	1
		UBE3A mut	1	1	/	0	1	0
Vocalic inventory								
			i	e	ɛ	a	ч	o
	Tonic	Deletion	/	/	1	7	/	/
		UBE3A mut	1	1	1	3	1	/
	Atonic	Deletion	/	/	/	2	/	/
		UBE3A mut	1	1	/	2	/	/

### Behavioural profile

All patients except one scored above the 95^th^ centile in the attention deficit scale. Seven subjects had scores higher than 95^th^ centile in hyperactive disorder scale, the other patients had scores between 50^th^–75^th^ and 90^th^–95^th^ centile. All except one had scores higher than 95^th^ centile in the total scale.

### Neurovisual evaluation

Table 5 summarizes neurovisual evaluation results. All the children presented refractive errors (most of them had hypermetropic astigmatism) and 5 had a hypopigmentation of fundus oculi. Strabismus (especially exotropia) was documented in six subjects, unstable visual fixation in three, discontinuous smooth pursuit in six and mild difficulties to perform voluntary saccades were observed in four children. None of the subjects presented visual perceptual deficit (visual acuity was near normal and all the subjects were able to locate targets presented in different areas of the visual field). Screening of visuocognitive abilities was not possible because of the severe intellectual delay of the whole sample, which also showed a moderate to severe visual attention impairment.

**Table 5:** Neurovisual features

Sbj	Refr. errors	Fundus oculi	Visual fixation	Smooth pursuit	Saccades	Strabismus	Abnormal ocular movement	Visual acuity	Contrast sensitivity (%)	Visual field	Visual attention
Pat. 1	A, I	Normal	Altered	Altered	Normal	Exotropia	No	6.3/10	1.25	Normal	Altered
Pat. 2	A, I	Normal	Normal	Normal	Normal	No	No	6.3/10	1.25	Normal	Altered
Pat. 3	A, I	Normal	Normal	Altered	Altered	Exo-hypotropia	No	13 cy/deg	1.25	Normal	Altered
Pat. 4	A, I	Normal	Normal	Altered	Altered	Esotropia	No	9.6 cy/deg	5	Normal	Altered
Pat. 5	A, I	Hypop.	Altered	Altered	Normal	No	No	9.6 cy/deg	1.25	Normal	Altered
Pat. 6	A, I	Hypop.	Normal	Altered	Normal	Exotropia	No	9.6 cy/deg	1.25	Normal	Altered
Pat. 7	A, I	Hypop	Altered	Altered	Altered	Exotropia	No	9.6 cy/deg	1.25	Normal	Altered
Pat. 8	A, M	Hypop.	Normal	Normal	Normal	Exotropia	No	9.6 cy/deg	1.25	Normal	Altered
Pat. 9	A, I	Normal	Normal	Normal	Altered	No	No	14 cy/deg	5	Normal	Altered
Pat. 10	A	Hypop.	Normal	Normal	Normal	No	No	14 cy/deg	1.25	Normal	Altered

*A* astigmatism, *I* hypermetropia, *M* myopia, *Hypop* hypopigmentation, *OD* optic disc, *Ny* latent nystagmus with horizontal jerky

## Discussion

This study has permitted to evaluate the neurodevelopmental aspects of a group of subjects with AS. Despite the small number of subjects, a high homogeneity of the results has been found among those evaluated, as literature data shows [4].

With reference to motor skills, a delay in acquiring motor milestones (head control, sitting and walking) has been documented. In particular, the acquisition of walking skills seems to be the most difficult milestone to reach, with significant differences between DEL and MUT groups. Ataxia has seemed to threaten the access to more complex GMFMS tasks, such as Standing and Walking ones. To our knowledge, no previous studies with GMFMS in AS children are reported in literature even if it could be considered an interesting standardized instrument to describe the motor functional disability in AS population. Clinicians should consider the opportunity to evaluate neuromotor abilities early, in order to promote at first, the achievement of motor milestones.

Cognitive assessment, proposed throughout psychometric tests and instruments which give a qualitative evaluation of cognitive competences, confirmed the severe intellectual disability as reported in literature [11, 12]. The psychometric evaluation by GMDS showed that none of sample reached 24 months of MA with a mean MA of 14.1 months, while the qualitative evaluation by UHS showed the achievement of ceiling effects in most of UHS subscales in 2 children out of 10. The presence of better performances in UHS scales than in GMDS can probably be explained by the different approaches to cognitive evaluation: most of the tasks included in psychometric instruments, as GMDS, are based on precise instructions that directly indicate what is expected. Responses typically have a verbal component, which is another factor that affects AS patients who cannot speak more than few words. In UHS scales, no direct indications are provided, but only the elicitation of behaviours as a consequence of the presentation of a stimulus.

As regards adaptive functions evaluated by VABS, communication and daily living skills were the most affected scales, as most of the subjects’ performance showed floor effect. MUT subjects showed better abilities especially in personal and gross motor skills.

With reference to language abilities, a severe impairment of receptive and expressive skills has been found at all ages. To our knowledge this is the first study that has identified the peculiar repertoire of word comprehension, phonemic inventory and referential gestures in AS.

Receptive competences could reach the second year of MA and were characterized in the entire sample, irrespective of the age, by a major comprehension level of those words that are typically understood even during the first stages by the Italian infant population [30].

The same trend has been identified in phoneme processing development: the entire sample can only acquire some nasal and occlusive phonemes, as it normally happens in the first steps of Italian phoneme acquisition [31].

Only some children with AS showed gesture competences such as referential and deictic gestures and none of them presented pretending play skills, which involves language use and takes place in social contexts. Expressive language speech wasn't present, despite the presence of cognitive precursors of language in 4 children and the presence of deictic gesture in 4.

These findings suggest the role of other factors which could be related to language development in AS. Penner and colleagues [32] refer respectively to stage 5 and 6 as a prerequisite for language and speech development. Even considering only the subjects who reached the sixth stage, none of them was able to speak more than five words. These results let us hypothesize that the lack of expressive language is not simply justified by the severe intellectual disability in AS population. Neither difficulties in interpersonal relationships seem to be the cause of expressive disorders since communicative inputs emerged from VABS, UHS and PVB. We could not administer ADOS scales because of the presence of MA lower than 12 months in 5 children.

Oller and colleagues [33] analyzed the importance of babbling as one of the prerequisites for later speech development. None of the subjects had normal babbling patterns, suggesting a massive impairment of the phono-articulation networks as a determinant cause of the lack of language production. Moreover, recent DTI Magnetic Resonances in AS have shown alterations in associated tracts and in particular in left arcuate fasciculus which reveals severe morphological changes, leading to hypothesize a generalized white matter alteration [34]. More studies are needed to better understand the mechanisms, which underline AS language competences impairment.

Our study demonstrates the importance of evaluating visual function in AS. The reduced pigmentation of fundus oculi can be explained by the haplo-insufficiency of OCA2 gene, which is involved in the development of pigment in the skin, hair, and eyes and located close to UBE3A gene [35].

Refractive errors (especially characterized by astigmatism associated with hypermetropia) were detected in all the subjects, esotropia was documented in one patient, exotropia in four and exo-hypotropia in one. To our knowledge, only one study reported the same results [20]. The presence of refractive errors in all the AS sample underlines the importance of an early neurovisual evaluation to detect the type of refractive errors and to prescribe optical devices. An early correction of refractive errors can ameliorate visual acuity for activities of daily living and finally can promote the development of cognitive functions, as has been documented in patients with other genetic syndromes [36]. The oculomotor abilities, evaluated from a qualitative point of view, were impaired in AS: fixation was unstable, smooth pursuit discontinuous and saccades mild impaired. The role of the oculomotor disorders is not clear but our previous experiences in other neurological pathologies documented a relation between oculomotor impairment and attention deficit [37]. Even if this relation is not clear and further studies on larger samples are necessary, an early identification of oculomotor disorders and early promotion of oculomotor functions may have a positive effect on attention deficit.

Visual perceptual abilities were difficult to evaluate because of children poor compliance. We can however infer, throughout the normal contrast sensitivity and the ability to locate targets presented in different areas of the visual field, that there were not any major visual perceptual problems. Moreover, using preferential looking procedure we observed a mild reduction in visual acuity that could be an expression of uncorrected refractive errors.

## Conclusions

This study represents one of the first attempts to analyze and integrate clinical aspects typical of AS, throughout the usage of qualitative and psychometric assessment tools.

The results demonstrate that AS clinical profile could be considered as a consequence of several aspects that interact each other and play a negative role on global development. Neurovisual problems, with particular regard to refractive errors and to oculomotor impairment should be taken into account at early stages of development because they could be related to and can later influence cognitive and attentional functions. Origin of the peculiar language disorders is not still clear and cannot be simply justified by the lack of cognitive prerequisites.

These data suggest that an early and careful assessment of motor, neurovisual, cognitive, linguistic and attention abilities should be essential for a correct identification of strengths and weaknesses of this clinical population so as to plan tailored rehabilitation programs, based on the promotion of motor milestones, neurovisual function and communication abilities during the first stages of life.

## Abbreviations

ADHD: Attention Deficit and Hyperactive Disorder
AS: Angelman syndrome
DEL: UBE3A deletion
GMDS: Griffiths Mental Developmental Scales
GMFMS: Gross Motor Function Measure Scale
MA: Mental age
MUT: UBE3A mutation
PVB: MacArthur-Bates Communicative Development Inventory
UHS: Uzgiris Hunt Scale
VABS: Vineland Adaptive Behaviour Scales
